# Association between fruit intake and abdominal adiposity in 1,707 randomly selected U.S. children

**DOI:** 10.3389/fnut.2025.1592654

**Published:** 2025-06-04

**Authors:** Larry A. Tucker

**Affiliations:** College of Life Sciences, 237 SFH, Brigham Young University, Provo, UT, United States

**Keywords:** epidemiology, energy density, dietetics, public health, abdominal obesity, waist

## Abstract

**Background:**

This investigation was conducted to determine the relationship between fruit intake and abdominal adiposity in 1,707 U.S. children.

**Methods:**

The children were randomly selected as part of the National Health and Nutrition Examination Survey (NHANES), so the sample represented U.S. children 8–11 years old. A cross-sectional design was employed. Fruit consumption was measured using the average of two 24-h dietary recalls. Fruit intake was expressed as the percent of total energy derived from fruit, not including fruit juices. Abdominal adiposity was indexed using two methods: waist circumference and the sagittal abdominal diameter (SAD). Covariates were age, sex, race, household size, year of assessment, recreational computer time, physical activity, total energy consumption, and intake of carbohydrate, protein, fat, fiber, sugar, and saturated fat. The outcome measures were waist circumference and sagittal abdominal diameter.

**Results:**

According to the findings, mean fruit consumption was 10.1% of total energy intake. With fruit intake and abdominal adiposity both treated as continuous variables, after controlling for all the covariates, there were significant inverse linear relationships between the log10 of fruit intake and waist circumference (*F* = 6.5, *p* = 0.0143) and SAD (*F* = 7.0, *p* = 0.0112). Similarly, with fruit consumption divided into 3 categories (None, Low, and Moderate/High), means of the abdominal adiposity variables differed across the fruit categories in a dose–response pattern (SAD: *F* = 3.4, *p* = 0.0407; Waist: *F* = 2.9, *p* = 0.0657), after adjusting for all the covariates.

**Conclusion:**

In this nationally representative sample of U.S. children, fruit consumption was low. Higher levels of fruit consumption were predictive of lower levels of abdominal adiposity. These findings support the recommendation of the U.S. Dietary Guidelines for Americans that encourage children to eat more fruit. Given the results, physicians, teachers, and parents should educate and encourage children about the importance of fruit consumption.

## 1 Introduction

In the United States, over 40% of children have overweight or obesity (1). In the past 50 years, the prevalence of overweight and obesity in children and adolescents has increased almost five-fold (2). Unfortunately, the risk of serious health conditions is much higher among youth who carry excess weight and body fat. Specifically, these individuals are more likely to have insulin resistance, high cholesterol, and high blood pressure, to name a few (1). They are also at increased risk of type 2 diabetes and cardiovascular disease early in their lives. Emotional issues such as depression and anxiety, as well as bullying and other social troubles, are also more likely in children with overweight or obesity (1).

The fact that overweight and obesity often lead to a multitude of health problems in children and adults is well-established. However, research indicates that the location of body fat is also a critical factor affecting disease risk. In their recent 2025 publication, the Lancet Diabetes & Endocrinology Commission stated that the commonly used body mass index (BMI) does not differentiate between fat and lean mass, and it does not account for differences in the distribution of body fat. The Commission declared that alternative measures, such as the waist circumference, might be more accurate for the detection of excess body fat and obesity-related health risks (2).

In a sample of 174 children from the United Kingdom, abdominal adiposity was a significant predictor of artery stiffness (3). In an investigation of 948 Saudi children, there were significant relationships between abdominal adiposity and numerous metabolic parameters (4). Labyak et al. (5) found that abdominal obesity in 145 youth was predictive of elevated triglycerides, HbA1c, systolic blood pressure, and a total risk score. Other investigations have also shown that abdominal adiposity is a good predictor of metabolic issues in children (6, 7).

The causes of childhood obesity and abdominal adiposity are multidimensional and complex. However, it is clear that diet plays a substantial role. Although there are many dietary components that contribute to excess weight and body fat, numerous investigations indicate that the energy density of food is an important factor (1, 8–12). Food energy density is the amount of energy in a food divided by its weight (kilocalories/grams). In short, the number of kilocalories (kcal) per gram of food. Some foods have very low energy densities, such as watermelon [0.30], strawberries [0.32], and peaches [0.39], whereas other foods have high energy densities, such as many candy bars [~4.8], bacon [5.3], and potato chips [5.4] (13).

The energy density of food is a function of the water content, the amount of fiber in the food, and the fat level (14). Foods that contain little water and fiber and have large amounts of dietary fat are energy dense. These items contain high amounts of energy but provide relatively little food to eat (14). Dietary patterns that include large amounts of energy dense foods often lead to excess body weight, whereas regular consumption of low energy dense foods tends to result in less obesity and abdominal adiposity (8, 9, 11, 12). Among the many foods available in the U.S., fruits tend to be among the lowest in energy density.

The relationship between fruit intake and obesity has been studied extensively in adults. For example, in a meta-analysis of prospective cohort studies, higher fruit intake was inversely and favorably associated with weight change in 17 of 20 investigations (15). In the classic Women’s Health Study, those in the highest quintile of baseline fruit intake had 13% less risk of having overweight or obesity compared to those in the lowest quintile (16). In a recent cross-sectional investigation, over 10,000 Korean adults were studied. Frequency of whole fruit intake was associated significantly and inversely with the prevalence of abdominal obesity (17). Similarly, in a large cross-sectional study of Peruvian adults, servings of fruit were negatively associated with central fat distribution (18).

Although the fruit consumption and adiposity relationship has been investigated many times in adults, research in children is less common. Moreover, few investigations have focused on the link between fruit intake and central adiposity in children, and even fewer studies have employed a large sample of children representative of U.S. youth. Therefore, the present investigation was conducted to evaluate the association between fruit intake and abdominal adiposity in a large random sample of U.S. children.

## 2 Methods

### 2.1 Study design and sample

The present cross-sectional investigation was conducted using data supplied by the U.S. National Health and Nutrition Examination Survey, NHANES. The survey is a government-sponsored project administered by the U.S. National Center for Health Statistics (NCHS) and the Centers for Disease Control and Prevention (CDC).

Written informed consent was obtained before data were collected by NHANES. The data gathering process and the data files were approved by the Ethics Review Board of the NCHS (19). The NHANES data files included no confidential information that could be connected to an individual. The data that support the findings of this study are available in NHANES Questionnaires, Datasets, and Related Documentation at https://wwwn.cdc.gov/nchs/nhanes/Default.aspx (20).

The data associated with the current investigation were gathered during a 6-year period, from 2011–2016. Other years were not included because the sagittal abdominal diameter variable was only measured during this time frame. The ethical approval code associated with these 6 years of data collection was Protocol #2011–17 (19).

NHANES employs a 4-step sampling procedure to randomly select participants using census data. Specifically, U.S. counties, then blocks or roads, then dwelling units, and lastly individuals were randomly selected. A total of 1,814 children, 8–11 years of age, were randomly selected. Children who reported extremely low energy intakes (< 400 kcal) on either of the two 24-h recall days were not included in the sample (*n* = 10). Children who were classified as underweight based on the age and sex-specific results of the CDC 2000 Growth Charts were not included in the sample due to concerns of an eating disorder or significant illness (*n* = 56). Also, children with missing data for one or more variables used in the present investigation were not included in the sample (*n* = 41). Hence, the final sample included 1,707 participants.

### 2.2 Measurement methods

Fruit intake was the exposure variable in this investigation. Abdominal adiposity, measured using two methods, was the outcome measure. Age, sex, race, household size, year of assessment, physical activity, recreational computer use, total energy intake, and consumption (grams per 1,000 kcal) of carbohydrate, protein, fat, sugar, saturated fat, and dietary fiber were the covariates.

#### 2.2.1 Fruit intake and other dietary variables

Fruit consumption and intake of 7 additional dietary variables were measured using the average of two 24-h dietary recall assessments. When appropriate, a parent or guardian assisted with the dietary assessment. The initial dietary evaluation was in-person. A telephone interview was employed to gather dietary information 3 to 10 days later for the second recall assessment. Mean values from the two recall assessments were used. Both 24-h recall assessments collected in-depth information about all foods and beverages consumed during the 24-h period before the interview (12:00 AM to 12:00 AM) (21). A computer provided a standardized interview outline. Scripts were employed to guide the interviewer (21). The Automated Multiple Pass Method (AMPM) guided the interviewer (22).

The in-person dietary interviews included several examples to help gauge the amount of food consumed, such as different sized plates, bowls, glasses, cups, spoons, etc. Following completion of the initial 24-h recall assessment, participants were furnished with sample glasses, cups, plates, etc. and a food model booklet to help during the subsequent telephone diet interview.

The focus of the present study was on fruit consumption, defined according to the U.S. Department of Agriculture (USDA) (13). Fruit juice was not included. Because larger individuals typically consume more total food and more food energy than smaller individuals, rather than focusing on the grams of fruit consumed, the present study concentrated on the percentage of total energy derived from fruit. Specifically, based on the weight of each fruit eaten during the two 24-h recall assessments, the energy value of each fruit was calculated. Then, the total energy intake from all fruits consumed was divided by the average total energy intake of the individual over the two dietary assessments, resulting in the percentage of total energy derived from fruit.

#### 2.2.2 Abdominal adiposity

Abdominal adiposity was indexed using two methods: waist circumference (waist) and sagittal abdominal diameter (SAD). Both are excellent measures of abdominal obesity and metabolic risk in adults (23–28) and children (4, 6, 29).

For the waist and SAD measurements, health technicians participated in 2 days of extensive training and periodic follow-up evaluations. The individual performing the measurements was assisted by a recorder. For the waist measurement, a wall mirror was also used to check the level of the measuring tape. The waist measurement was calculated to the nearest 0.1 cm after the individual exhaled a normal breath. Detailed measurement methods are available online (30).

The sagittal abdominal diameter (SAD) is an index of abdominal height with the participant in the supine position on an examination table. SAD was assessed by using a sliding-beam, abdominal caliper (Holtain, Ltd., Wales, UK). A second SAD measurement was always taken, and the mean was calculated. If the SAD measurements differed by more than 0.5 cm, then a third and possibly a fourth measurement was taken, and the mean was calculated (30). SAD intra-tester precision is excellent (31). The measurement method for assessing SAD is explained in detail online (30).

#### 2.2.3 Covariates

NHANES recorded sex as male or female based on the sex recorded at birth. Race was recorded as non-Hispanic Black, non-Hispanic White, Mexican American, Other race/multiracial, or Other Hispanic. Household size was the number of individuals currently living in the dwelling unit, truncated at 7. Year of assessment was based on the NHANES 2-year data collection cycles. SAD data were only collected by NHANES from 2011 to 2016. Physical activity was the number of days during the past week the child participated in physical activity for an hour or more. Time spent in recreational computer use was the average number of hours spent on a computer each day over the past 30 days, outside of work or school. Information about the dietary covariates was collected as part of the two 24-h dietary recall assessments.

### 2.3 Data analysis

Because NHANES utilizes a unique, multi-stage random sampling procedure to select participants, statistical procedures involving NHANES data require special procedures. Specifically, each statistical analysis of NHANES data must be weighted so that the results can be generalized to the U.S. population. Additionally, even though the number of children in this investigation was large (*N* = 1,707), the unique sampling procedure employed by NHANES resulted in a significant reduction in the degrees of freedom (df) available. Specifically, df equaled the number of clusters [91] minus the number of strata [44], resulting in 47 df, instead of approximately 1,707 df. Therefore, statistical power was much lower than the sample size would typically dictate.

There were two outcome variables, SAD and waist circumference, both measures of abdominal adiposity. The exposure variable was the percentage of total energy derived from fruit. Five demographic covariates were controlled, age, sex, race, year of assessment, and household size. Additionally, physical activity, recreational computer use, and numerous dietary measures were the covariates.

To assess the associations between the exposure variable, fruit intake, and the outcome variables (waist and SAD), two different analyses were performed. First, both fruit intake and the outcome variables were treated as continuous variables. This allowed the linear association to be evaluated using the SAS SurveyReg procedure. Because the fruit intake variable was skewed and more than 1 in 4 participants reported no energy derived from fruit, the log10 of the fruit intake variable was used as the exposure variable. Because the log10 of 0 cannot be calculated, 0.1 was used instead of 0, resulting in a substantial reduction in the skewness of the distribution.

Second, fruit intake was treated as a categorical variable and mean differences in abdominal adiposity (SAD and waist) were compared across the fruit consumption categories using SAS SurveyReg. There were three fruit intake categories: None, Low, and Moderate/High. Children in the “None” category had zero fruit intake. Youth in the Low category consumed some fruit but less than 9.2% of their total energy intake was derived from fruit. Children in the Moderate/High category consumed at least 9.2% of their energy from fruit. The Low and Moderate/High fruit groups were constructed to be equal in size. Partial correlation and the LSmeans procedure were used to control differences in the covariates and to compare the adjusted means to determine if they differed significantly.

Effect modification was tested to determine the extent that the fruit intake and the abdominal adiposity relationships were consistent within subgroups. SAS version 9.4 (SAS Institute, Inc., Cary, NC) was utilized to examine relationships of interest. Alpha was set at < 0.05 to establish significance. Marginal significance was *p* > 0.05 and *p* < 0.10. The statistical tests were all two-sided.

## 3 Results

From 2011 to 2016, there were 44 primary sampling units (PSU) selected randomly by NHANES in the U.S. From these PSU, 91 clusters were randomly selected. A total of 1707 randomly selected children were included in the sample. They represented the population of 8–11-year-old children in the U.S. Mean (±SE) age, SAD, and waist of the sample were 9.5 ± 0.04 years, 15.4 ± 0.12 (cm), and 68.5 ± 0.44 (cm), respectively. Average (±SE) number of days per week that the children engaged in at least 1 h of physical activity was 5.7 ± 0.07 and mean (±SE) recreational computer use per day was 1.2 ± 0.05 h. Average (±SE) fruit intake per day was 10.1 ± 0.6% of total energy intake. Table 1 shows the percentile distributions associated with each of the continuous measures included in the investigation.

**Table 1:** Percentile distributions of the continuous variables representing U.S. children, (*n* = 1,707).

Variable	Percentile				
	10th	25th	50th	75^th^	90th
Age (years)	8.0	8.0	9.0	10.0	10.6
Sagittal abdominal diameter (cm)	12.4	13.3	14.6	16.8	19.1
Waist circumference (cm)	56.2	59.6	65.6	74.7	84.5
Fruit intake (percent of total kcal)	0	0	5.4	14.7	27.2
Fat intake (grams per 1,000 kcal)	30.0	33.7	37.6	41.3	44.9
Carbohydrate intake (grams per 1,000 kcal)	112.7	122.2	132.8	141.3	151.9
Protein intake (grams per 1,000 kcal)	26.2	30.5	35.4	40.5	46.9
Sugar intake (grams per 1,000 kcal)	40.3	49.5	59.6	69.7	80.3
Saturated fat intake (grams per 1,000 kcal)	9.4	11.2	13.1	15.2	17.1
Fiber intake (grams per 1,000 kcal)	5.1	6.1	7.4	9.1	11.3
Energy intake (kilocalories per day)	1,351	1,620	1909	2,309	2,704
Physical activity (days/week of 1 h or more)	2.2	4.2	6.1	6.6	6.8
Recreational computer use (hrs/day)	0	0	0.4	1.4	2.7

Table values include person-level weighted adjustments based on the sampling methods of NHANES, so values represent those of the U.S. population of children 8–11 years old. Fruit intake (percent of total kcal) refers to the percent of the total kilocalories derived from fruit, not counting fruit juices.

For the categorical variables, which are shown in Table 2, there was almost an even split between girls and boys. The race distribution differed slightly from the U.S. population because NHANES intentionally oversampled Mexican Americans. One-third of the sample had households with four individuals and about one-fourth of the sample included households with a total of five individuals. Almost 27% of the sample reported no fruit intake, and the remaining 73% were intentionally divided evenly into two categories.

**Table 2:** Characteristics of the sample based on the categorical variables (*n* = 1,707).

Categorical variable	*N*	%	SE
Sex			
Girls	827	48.5	2.14
Boys	880	51.5	2.14
Race/ethnicity			
Non-Hispanic White	867	50.8	3.38
Non-Hispanic Black	244	14.3	1.94
Mexican American	303	17.8	2.44
Other race/multiracial	157	9.1	1.14
Other Hispanic	136	8.0	0.98
Household size			
2	54	3.0	0.57
3	211	12.0	1.10
4	585	33.2	1.82
5	454	25.8	1.59
6	275	15.6	1.29
7 or more	183	10.4	0.97
Year of assessment			
2011–2012	567	33.2	2.39
2013–2014	555	32.5	3.05
2015–2016	585	34.3	2.61
Fruit intake			
None	456	26.7	2.14
Low	623	36.5	2.00
Moderate/high	628	36.8	1.81

Table values include person-level weighted adjustments based on the sampling methods of NHANES, so values represent those of the U.S. population of children.

The exposure variable was fruit intake, expressed as a percentage of the total energy consumed. As shown in Table 3, there was a significant linear relationship between fruit intake (normalized using log10) and SAD (df = 47), after adjusting for the demographic covariates (Model 1: *F* = 6.4, *p* = 0.0148), after adjusting for the demographic and lifestyle covariates together (Model 2: *F* = 7.0, *p* = 0.0110), and after controlling for the demographic, lifestyle, and additional dietary covariates, including intake (grams per 1,000 kcal) of carbohydrate, protein, fat, sugar, saturated fat, and dietary fiber (Model 3: *F* = 7.0, *p* = 0.0112). The association between fruit intake and waist circumference (df = 47) was also inverse, linear, and significant across all three statistical models (Table 3).

**Table 3:** Regression analysis showing the linear relationship between fruit intake and abdominal adiposity after adjusting for different covariates.

*N* = 1,707				
Exposure variable: fruit intake	Regression coefficient	SE	*F*	*p*
Sagittal abdominal diameter (cm)				
Model 1	−0.20	0.08	6.4	0.0148
Model 2	−0.21	0.08	7.0	0.0110
Model 3	−0.26	0.10	7.0	0.0112
Waist Circumference (cm)				
Model 1	−0.89	0.36	6.2	0.0162
Model 2	−0.91	0.35	6.8	0.0122
Model 3	−1.03	0.40	6.5	0.0143

SE: standard error of the regression coefficient. Fruit intake was expressed as the percent of total energy derived from fruit, not counting fruit juices. For Model 1, the covariates were age, sex, race, year of assessment, and household number. For Model 2, in addition to the Model 1 covariates, adjustments were made for differences in physical activity, recreational computer time, and mean energy intake. For Model 3, in addition to the Model 2 covariates, differences in the intake (grams per 1,000 kcal) of carbohydrate, protein, fat, sugar, saturated fat, and fiber were controlled statistically. Because the fruit intake distribution was skewed, the log10 of fruit intake was used. Table values included person-level weighted adjustments based on the sampling methods of NHANES, so values represented those of the U.S. population of children, 8–11 yr.

In Table 3, both fruit consumption and abdominal adiposity (SAD and waist) were treated as continuous variables. However, in Table 4, abdominal adiposity was expressed as a continuous variable and fruit intake was treated as a categorical variable. In Table 4, with fruit intake divided into 3 categories, again there was a dose–response relationship. With all the covariates controlled, SAD (abdominal height) was significantly smaller for the children who consumed a moderate to high percentage of their energy from fruit compared to children who ate no fruit. Likewise, waist circumferences tended to be smaller for youth who ate a moderate to high percentage of energy from fruit compared to children who ate no fruit. In each case, the associations were inverse and dose–response.

**Table 4:** Mean differences in abdominal adiposity, indexed using waist circumference and SAD, across categories of fruit intake in U.S. children, after adjusting for the covariates.

Outcome variable	Fruit intake (percent of energy intake from fruit)							
	None (*n* = 456)		Low (*n* = 623)		Moderate/High (*n* = 628)			
	Mean	± SE	Mean	± SE	Mean	± SE	*F*	*p*
SAD (cm)								
Model 1	15.9^a^	0.15	15.6^b^	0.16	15.4^b^	0.16	3.9	0.0283
Model 2	15.9^a^	0.18	15.5^b^	0.18	15.4^b^	0.21	4.0	0.0252
Model 3	15.9^a^	0.20	15.6^b^	0.18	15.3^b^	0.21	3.4	0.0407
Waist (cm)								
Model 1	70.5^a^	0.65	69.0^b^	0.70	68.4^b^	0.65	3.5	0.0380
Model 2	70.2^a^	0.64	69.4^a^	0.65	68.1^b^	0.65	7.7	0.0013
Model 3	70.4^a^	0.80	68.9^a^	0.82	68.0^b^	0.84	2.9	0.0657

SAD, sagittal abdominal diameter. There were 47 df in the denominator of each model. Fruit intake was indexed as the percent of total energy derived from all fruits consumed, not including fruit juices. Means on the same row with the same superscript letter were not significantly different. Model 1 included the following covariates: age, sex, race, year of assessment, and household size. Model 2 included the Model 1 covariates and also hours of recreational computer time, physical activity, and average energy intake. Model 3 included the Model 2 covariates and also intake (grams per 1,000 kcal) of carbohydrate, protein, fat, fiber, sugar, and saturated fat. For SAD, the difference between the no fruit and low intake groups were marginally significant. For Waist, Model 1, the no fruit intake vs low, and for Model 2, the low vs moderate/high intake groups, were marginally significant. For Waist, Model 3, the relationship was marginally significant (*p* = 0.0657). Table values included person-level weighted adjustments based on the sampling methods of NHANES, so values represented those of the U.S. population of children, 8–11 years old.

To test the extent that fruit intake was related to SAD and waist circumference within specific subgroups, gender, race and ethnicity, recreational computer use, and physical activity were divided into meaningful categories. The results are shown in Table 5.

**Table 5:** The relationship between fruit intake, sagittal abdominal diameter, and waist circumference within key subgroups.

Exposure variable: fruit intake	Sagittal abdominal diameter (cm)			
	Regression Coefficient	SE	*F*	*p*
Gender				
Boys only	−0.23	0.10	5.3	0.0253
Girls only	−0.16	0.14	1.3	0.2616
Race and ethnicity				
Non-Hispanic White only	−0.23	0.13	3.2	0.0804
Non-Hispanic Black only	−0.06	0.16	0.1	0.7129
Mexican American only	−0.34	0.22	2.3	0.1342
Non-White only	−0.18	0.08	4.8	0.0342
Non-Black only	−0.21	0.09	5.3	0.0259
Non-Mexican American only	−0.19	0.08	5.6	0.0226
Computer use				
0–2 h per day only	−0.22	0.08	7.1	0.0106
≥ 3 h per day only	−0.10	0.22	0.2	0.6612
Physical activity				
0–3 days per week only	−0.09	0.33	0.1	0.7934
4–7 days per week only	−0.21	0.09	5.6	0.0219
Waist circumference (cm)				
Gender				
Boys only	−0.84	0.48	3.1	0.0845
Girls only	−0.93	0.55	2.9	0.0967
Race and ethnicity				
Non-Hispanic White only	−1.02	0.55	3.5	0.0681
Non-Hispanic Black only	−0.25	0.72	0.1	0.7335
Mexican American only	−1.16	0.92	1.6	0.2165
Non-White only	−0.77	0.34	5.0	0.0303
Non-Black only	−0.94	0.41	5.2	0.0269
Non-Mexican American only	−0.89	0.36	6.0	0.0179
Computer use				
0–2 h per day only	−1.03	0.37	7.6	0.0085
≥ 3 h per day only	−0.21	1.06	0.0	0.8453
Physical activity				
0–3 days per week only	−0.66	1.47	0.2	0.6544
4–7 days per week only	−0.88	0.38	5.3	0.0256

Because the distribution of fruit intake was skewed, the log10 of fruit intake was used. Non-Mexican Am., Non-Mexican American. Table values included person-level weighted adjustments based on the sampling methods of NHANES, so values represented those of the U.S. population of children, 8–11 years old.

According to Table 5, the relationship between fruit intake and SAD was significant for boys but not for girls. For waist size, the association was marginally significant for both girls and boys considered separately. For subgroups based on race and ethnicity, the abdominal adiposity measures were significant or marginally significant for all the subgroups except Non-Hispanic Black and Mexican Americans. For subgroups based on recreational computer use, the fruit intake and abdominal obesity associations were inverse and significant for youth who kept their recreational computer use to 2 h or less per day. However, the relationship was not significant for youth who spent 3 h per day or more in recreational computer use. Similarly, based on subgroups of physical activity, the association between fruit intake and abdominal adiposity was inverse and significant in youth who engaged in at least 1 h of physical activity 4 days per week or more, but the relationship was not significant in youth who engaged in less than 4 days per week of physical activity.

Table 6 shows the extent that the dietary and other covariates differed across the 3 fruit intake categories. Fiber intake had the strongest relationship with fruit intake (*F* = 62.8, *p* < 0.0001). The association was dose–response. Those in the highest fruit intake category consumed 37% more fiber than those in the no fruit intake category.

**Table 6:** Mean differences in dietary and other variables across the fruit intake categories.

Outcome variable	Fruit Intake (percent of energy intake from fruit)							
	None (*n* = 456)		Low (*n* = 623)		Moderate/High (*n* = 628)			
	Mean	± SE	Mean	± SE	Mean	± SE	*F*	*p*
Diet								
Fat intake (g/1000 kcal)	38.7^a^	0.44	37.9^a^	0.28	36.0^b^	0.32	19.6	<0.0001
Saturated fat (g/1000 kcal)	13.4^a^	0.22	13.4^a^	0.19	12.6^b^	0.22	8.2	0.0009
Protein intake (g/1000 kcal)	36.6	0.57	37.1	0.55	37.0	0.44	0.3	0.7331
Carb. intake (g/1000 kcal)	128.5^a^	1.04	130.1^a^	0.89	135.9^b^	0.75	27.3	<0.0001
Sugar intake (g/1000 kcal)	56.3^a^	1.04	56.9^a^	0.79	63.0^b^	0.79	18.6	<0.0001
Fiber intake (g/1000 kcal)	6.7^a^	0.14	7.7^b^	0.12	9.1^c^	0.17	62.8	<0.0001
Other variables								
Age (years)	9.5	0.06	9.5	0.07	9.5	0.07	0.1	0.9454
Computer use (hrs per day)	1.4	0.09	1.2	0.09	1.2	0.07	0.7	0.5085
Physical activity (days/wk)	5.6	0.15	5.8	0.11	5.5	0.10	1.7	0.1928
Household size	4.9	0.09	4.9	0.07	4.7	0.07	1.8	0.1710

Carb., carbohydrate. There were 47 degrees of freedom in the denominator of each model. Fruit intake was indexed as the percentage of total energy derived from all fruits consumed, not including fruit juices. Means on the same row with the same superscript letter were not significantly different. For each relationship, statistical adjustments were made for differences in the demographic covariates: age, sex, race, family size, and year of assessment, except when the covariate was studied as the outcome variable (age and household size). For example, when age was the outcome variable, age was not used as a covariate.

Carbohydrate and sugar consumption were also higher in the children reporting the most fruit intake compared to children with lower intakes of fruit. The association between dietary fat consumption and fruit intake was also strong (*F* = 19.6, *p* < 0.0001). The relationship was dose–response. Children with the highest intakes of fruit also had the lowest intakes of saturated fat (*F* = 8.2, *p* = 0.0009). The only dietary variable that was not related to fruit intake was protein consumptioin (*F* = 0.3, *p* = 0.7331).

Other covariates and their associations across the fruit intake categories included age, recreational computer use, physical activity, and the number of individuals in the household. Mean ages were identical across the 3 fruit categories. Moreover, there were no mean differences in the other covariates across the fruit intake categories.

## 4 Discussion

The main focus of this investigation was to determine the relationship between fruit intake and abdominal adiposity, indexed using SAD and waist circumference, in 1707 randomly selected U.S. children 8–11 years old. There were four key findings resulting from this study: (1) children consumed only 5–6% of their total energy from fruit (median), not including fruit drinks. (2) With fruit intake and abdominal adiposity both treated as continuous variables, the associations were linear, inverse, and significant. (3) With fruit consumption divided into 3 categories (None, Low, and Moderate/High), children with moderate to high fruit intake had less abdominal adiposity (both SAD and waist circumference) than children who did not consume fruit. (4) The association between fruit intake and abdominal adiposity was stronger and more consistent in U.S. boys than in girls.

The relationship between fruit intake and adiposity has been evaluated many times in adults. Hebden et al. (32) published a review that included 17 studies. The findings showed that fruit intake (not including juice) reduced the risk of long-term weight gain. The authors concluded that their review affirmed the U.S. Dietary Guidelines, which encourage Americans to consume more fruit. Although the review showed a consistent, inverse relationship between fruit intake and body weight in adults and adolescents, it did not include children as participants.

The relationship between fruit intake and adiposity has been studied infrequently in children compared to adults, and findings in children have been mixed. In a large prospective study of approximately 15,000 children and adolescents, Field et al. determined that fruit consumption was not predictive of changes in BMI (33). Similarly, in a prospective study by Bayer et al. (34), over 1,200 children who were about 6 years old at baseline were followed for 4 years. Gains in BMI were lower in children who increased fruit consumption over the 4 years, but the reduced gains were not statistically significant.

On the other hand, in a study by Wall et al. (35) of almost 200,000 adolescents who completed a food frequency questionnaire, those who reported eating fruit once or twice per week and those who reported consuming fruit three of more times per week had significantly lower BMIs than those who never ate fruit. Finally, Wang et al. (36) conducted a meta-analysis using only RCT. They found that fruit intake had no effect on BMI-z scores in children. However, the interventions decreased waist circumference in three of the four investigations that assessed waist circumference. None of the studies included a measure of sagittal abdominal diameter. It appears that abdominal adiposity may be a more sensitive measure of overweight and obesity than BMI or BMI-z in children.

In the present study of U.S. children, as fruit intake increased, abdominal adiposity decreased. The potential mechanisms accounting for this relationship are many and varied. However, one likely mechanism is the low energy density of most fruits because fruits typically contain large amounts of water, some fiber, and little or no dietary fat. Consequently, fruits tend to provide little food energy for their weight (14). Therefore, individuals can eat relatively large amounts of fruit without consuming a large amount of energy (14). Additionally, fruits are good sources of many vitamins, minerals, and bioactive compounds, such as carotenoids and phytochemicals.

Numerous investigations indicate that dietary patterns that include a large number of low energy dense foods, such as fruits, discourage over-consumption of energy and can assist with the management of body weight. For example, in a 2023 study by Diktas et al. (37), at snack time, 52 children consumed a larger proportion of self-served strawberries than pretzels, but because of the energy density difference between the foods, the children derived 55 ± 4 kcal more from the pretzels than the strawberries (*p* < 0.0001). In short, although the children ate more of the lower-energy dense strawberries, they consumed more energy from the higher-energy dense pretzels, emphasizing the impact of energy density in children’s energy intake.

In another study, Smethers et al. (38) employed a crossover design to determine the effect of varying the energy density of three main dishes and 1 snack per day for 5 days on 49 children. At baseline, the children’s energy intakes were consistent with their daily needs. However, serving higher energy dense foods resulted in higher energy intakes by 84 kcal per day, and serving lower energy dense foods resulted in lower energy intakes by 72 kcal per day (both *p* < 0.0001) over the 5 days. Adjusting the energy density of the foods had a meaningful and consistent impact on the children’s daily energy intakes (38).

In 2023, Rolls et al. (39) performed a secondary analysis based on the weighed intakes of 6,355 meals served to 94 children. The energy consumed at the meals was significantly related to the energy density of the consumed foods (*p* < 0.0001). Additionally, Leahy et al. employed a crossover design and evaluated a test lunch once per week for 6 weeks in children (40). Reducing the energy density of the entrée by 30% decreased the kcal consumption from the entrée by 25%. Although the children consumed more of the lower energy dense entrée, the children ate fewer kcal (40).

Guyenet (41) reported that randomized controlled trials (RCT), particularly those of high quality, show that fruit intake promotes weight maintenance or weight loss over time, and that high intake of fruit promotes weight loss. The review also suggested that single-meal RCTs show that fruit consumption tends to decrease kcal intake.

Another potential mechanism that could account for some of the association between fruit intake and abdominal adiposity in U.S. children is dietary fiber consumption. As shown in Table 6, in the current investigation, there was a very strong association between fruit and fiber intake. Children in the highest category of fruit consumption ate about 36% more fiber when compared to youth who reported no fruit consumption. Many studies in the literature have reported strong relationships between dietary fiber intake and reduced weight gain, lower body fat, and other body composition outcomes (42–45).

Numerous investigations over the past few decades have studied the impact of gut bacteria on obesity, body weight, and health (46–49). Given consumption of fruit and dietary fiber tend to have positive effects on the gut microbiome (46), it is reasonable that participants in the present study who consumed the most fruit and fiber had healthier gut microbiomes. This could be a mechanism accounting for the lower abdominal adiposity in those with higher fruit and fiber intakes. In short, increased fruit and fiber consumption may have resulted in less abdominal adiposity in the children because of their healthier gut microbiomes (46–49).

Differences in dietary fat intake could be another mechanism accounting for the inverse relationship between fruit intake and abdominal obesity. As displayed in Table 6, there was a substantial, inverse relationship between fruit intake and fat consumption (*F* = 19.6, *p* < 0.0001). Children who reported not eating fruit ate about 8% more dietary fat than youth with moderate to high intakes of fruit. Numerous studies indicate that as dietary fat increases in the diet of individuals eating ad libitum, body weight and obesity tend to increase (44, 50, 51). Moreover, according to the results of 32 randomized controlled trials reviewed by Hooper et al. (52), almost all the studies showed that eating less dietary fat results in greater weight loss than higher fat intakes. The increased dietary fat intake among youth eating less fruit could account for some of the differences in abdominal adiposity found in the present investigation.

Another mechanism that could help explain the inverse association between fruit intake and abdominal adiposity in children is the substitution effect. When children eat fruit, they are less likely to consume high calorie snacks, such as chips, cookies, and soft drinks. Over time, this could account for reduced energy consumption and less abdominal obesity. For example, a longitudinal study of 1,339 children in Norway found that 15 schools participating in a free fruit program saw a significant decrease in children’s intake of unhealthy snacks compared to children in 12 schools that did not participate. Specifically, the frequency of unhealthy snack consumption decreased from 6.9 to 4.6 times per week in the schools that participated in the free fruit program compared to the schools that did not participate (53).

Lastly, fruits are rich in vitamins, antioxidants, flavonoids, and phytochemicals that tend to reduce inflammation, as shown in a review and meta-analysis of 83 studies by Hosseini et al. (54). Overall, the meta-analysis revealed that circulating C-reactive protein and tumor necrosis factor alpha were improved by fruit and also vegetable consumption. Similarly, in a dietary study of adolescents, higher levels of fruit intake were associated with lower levels of C-reactive protein, interleukin-6, and urinary F_2_-isoprostanes, indicative of decreased inflammation and oxidative stress (55). Decreasing inflammation tends to increase insulin sensitivity and normalize hunger hormones, such as leptin and ghrelin, potentially improving weight management (56–58).

According to the 2020–2025 Dietary Guidelines for Americans, about 80% of the U.S. population do not meet the fruit consumption recommendations (1). The Guidelines indicate that most individuals would benefit from adding more fruit to their diet, particularly whole fruits in nutrient-dense forms. The U.S. Guidelines indicate that individuals should eat more whole fruits as snacks and also consume fruit as part of meals. Given the U.S. Guideline recommendations and the findings of this study, healthcare professionals, educators, and parents should encourage children to consume more fruit.

The current study was not without flaws. The most significant weakness was that a cross-sectional design was employed. Hence, conclusions pointing toward causation are not justified. However, it should be noted that randomized controlled trials have a significant weakness in that participants often do not eat the diet they are prescribed. In the present study, no diet was prescribed, so compliance, or lack of compliance, was not an issue. Participants simply reported what they ate, so the study focused on typical, unmanipulated intake. Another weakness could be that children who ate large amounts of fruit may be unique and have other characteristics that encourage less abdominal adiposity. Age, sex, race, year of assessment, household size, physical activity, recreational computer time, average energy intake, and consumption of carbohydrate, protein, fat, sugar, fiber, and saturated fat were controlled statistically to minimize this issue, but there are always unmeasured factors that could influence the results. Moving forward, scientists should focus on conducting prospective cohort studies and intervention studies to further clarity the causal link between fruit consumption and abdominal obesity.

The study also had multiple strengths. Participants were randomly selected from the U.S. population. Consequently, the results can be generalized to the U.S. population of children 8–11 years old. Additionally, the sample included nearly an equal number of girls and boys and all major races and ethnic groups within the U.S., so the findings fit a diverse population. Furthermore, the variables were measured using high-quality methods, and highly trained technicians collected the data, independent of the present investigation, so there were no experimental biases influencing the measurements. Also, a total of 14 potentially confounding variables were measured and controlled statistically. Finally, the sample was large (*n* = 1,707), providing stable results.

## 5 Conclusion

U.S. children tend to consume significantly less fruit than the U.S. Dietary Guidelines for Americans (2020–2025) recommend. In this investigation, as fruit consumption increased, abdominal adiposity, specifically the sagittal abdominal diameter and the waist circumference, decreased in U.S. children. The associations were inverse and linear, even after adjusting for differences in many potential confounding variables. Low energy dense fruits allow children to consume more food without consuming more energy. Fruits are also rich in vitamins, antioxidants, flavonoids, and phytochemicals, which may increase insulin sensitivity, and potentially help to normalize hunger-related hormones and promote other health benefits. Although causal conclusions are not warranted, it appears that abdominal adiposity in U.S. children is partly accounted for by different levels of fruit intake. Results from the present investigation align with the U.S. Dietary Guidelines which recommend that American children should eat more fruit. Consequently, healthcare professionals, teachers, and parents should encourage children to consume multiple servings of fruit each day.

## Data Availability

The datasets presented in this study can be found in online repositories. The names of the repository/repositories and accession number(s) can be found below: The data that support the findings of this study are openly available in NHANES Questionnaires, Datasets, and Related Documentation at: https://wwwn.cdc.gov/nchs/nhanes/Default.aspx.
